# Chk1 suppressed cell death

**DOI:** 10.1186/1747-1028-5-21

**Published:** 2010-09-02

**Authors:** Mark Meuth

**Affiliations:** 1 Institute for Cancer Studies, University of Sheffield, School of Medicine and Biomedical Sciences, Sheffield S10 2RX, UK

## Abstract

The role of Chk1 in the cellular response to DNA replication stress is well established. However recent work indicates a novel role for Chk1 in the suppression of apoptosis following the disruption of DNA replication or DNA damage. This review will consider these findings in the context of known pathways of Chk1 signalling and potential applications of therapies that target Chk1.

## Review

The orderly and accurate replication of DNA is essential for the maintenance of genetic stability in cells. Cell cycle checkpoints play a critical role in this process by sensing DNA damage or aberrant replication structures and then slowing entry into S-phase, progression through S-phase, and mitosis to facilitate repair [1]. Recent work from a number of laboratories shows that some of these checkpoint responses regulate apoptosis in response to disruptions of DNA replication. Cell death helps maintain genetic stability by eliminating damaged cells that are not likely to be repaired. Thus a balance between cell cycle arrest and repair and the induction of cell death that is determined by checkpoints is critical for the preservation of genetic integrity. p53 plays a major role in the induction of apoptosis through the transcriptional activation of proapoptotic genes such as BAX and PUMA in response to DNA damage [2-4]. Thus in tumour cells deficient in p53 the balance between cell death and cell cycle arrest/repair is compromised. If repair is incomplete or inaccurate, genetic abnormalities may accumulate in p53-deficient tumour cells in part because cells acquiring DNA damage are no longer committed to death. Recently a number of laboratories using very different approaches have attempted to restore apoptotic responses in tumour cells to make them more responsive to therapeutic agents. Intriguingly several of these efforts have become focused on the checkpoint kinase, Chk1, as being particularly critical for the control of apoptosis in tumour cells. Here I will review recent work implicating Chk1 as a key mediator of death in tumour cells in response to the disruption of DNA replication.

### The ATR-Chk1 pathway primarily responds to ssDNA generated by DNA replication stress

Chk1 is critical to a wide range of responses to DNA replication stress and some forms of DNA damage. Chk1 is rapidly phosphorylated at several sites in an Ataxia telangiectasia mutated and Rad3 related (ATR)-dependent manner after inhibition of DNA replication  [5]. These post translational modifications are required to trigger cell cycle checkpoints in S and G2 [6], suppress inappropriate firing of late or cryptic DNA replication origins [7], and maintain replication fork integrity [8,9]. The roles of Chk1 in cell cycle checkpoints overlap with those of another DNA damage response pathway controlled by the Ataxia telangiectasia mutated (ATM) protein and its downstream phosphorylation target Chk2. For example, both Chk1 and Chk2 phosphorylate Cdc25A targeting it for degradation by ubiquitin-mediated proteolysis [6,10]. In the absence of Cdc25A the Cdk2/cyclinE/A complex is inactive and S-phase arrest ensues. However the two pathways respond to different signals: single stranded DNA (ssDNA) for ATR-Chk1 and DNA double stranded breaks (DSBs), DNA double stranded ends, or collapsed replication forks for ATM-Chk2.

Although an expanding family of proteins appears to be necessary for Chk1 activation, much work implicates ssDNA generated by the inhibition of DNA replication as being critical for the ATR-mediated activation of Chk1. Replication progression is driven by the coordinated action of the replication helicase and DNA replication complexes. Work with *Xenopus laevis *egg extracts indicates that when replication is inhibited, the unwinding of DNA continues while the replication complex is stalled leading to the generation of ssDNA [11]. This ssDNA is rapidly coated by replication protein A (RPA) and the resulting RPA-ssDNA complex recruits ATR through the ATR interacting protein (ATRIP) [12]. The heterotrimeric Rad9-Rad1-Hus1 (9-1-1) DNA clamp is then loaded onto ssDNA regions by the Rad17-RFC2-5 complex [13,14] and TopBP1 is recruited to the stalled forks through its interactions with the 9-1-1 and ATR-ATRIP complexes [15,16]. Other proteins (including Claspin [17,18], Brit1/Mcph1 [19], and FANCM/FAAP24 [20]) have been reported to be essential for the efficient activation of Chk1. Still further proteins (including FancJ [21] and Tim-Tipin [22]) appear to influence Chk1 activation through their roles in the generation of ssDNA. *In vitro *biochemical studies of potential substrates for the initial DNA binding reactions using static heteroduplex DNAs and *Xenopus *egg extracts show that 5'ssDNA-dsDNA junctions arising on either leading or lagging strands are most effective for Chk1 activation [23,24]. Thus these studies implicate primed ssDNA at stalled replication forks as being an effective substrate for the activation of this damage response pathway in this model system.

ssDNA generated during the repair of some types of DNA damage also triggers Chk1 activation. End processing at ionizing radiation (IR)-induced DNA DSBs by the Mre11-Rad50-NBS1 complex that is initiated by ATM can generate ssDNA and activate Chk1 [25].

### Cellular consequences of loss of Chk1

**Figure 1:**
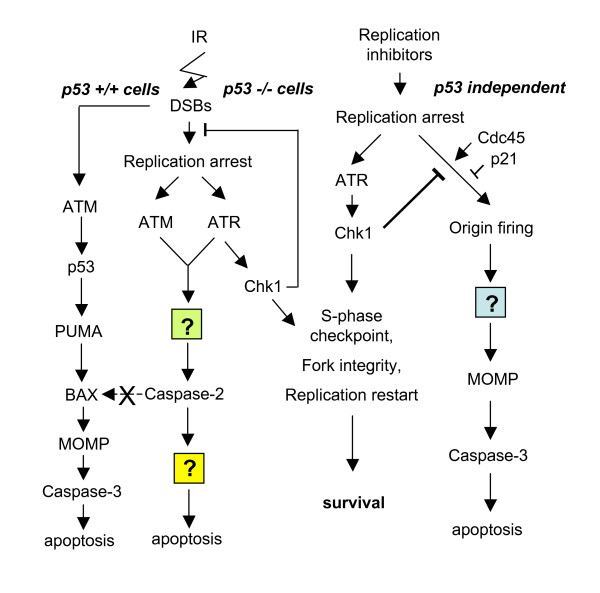
**Chk1 suppressed death pathways**. Chk1 responds to DNA replication stress in an ATR-dependent manner to trigger S-phase checkpoints, suppress inappropriate firing of late or cryptic DNA replication origins, and maintain replication fork integrity. When this ATR-Chk1 signalling pathway is suppressed cells show an enhanced level of apoptosis that appears to be the result of loss of control of replication origin firing [37]. This death pathway is characterized by mitochondrial outer membrane permeabilization (MOMP) and caspase-3 activation but is independent of p53 status [30]. The induction of apoptosis in p53 proficient cells is strongly ATM/p53-dependent. This death pathway characterized by the p53-dependent induction of the proapoptotic proteins PUMA and BAX, MOMP, and caspase-3 activation. p53 deficient cells have a much reduced death response following exposure to IR due to the protective effects of ATM- or ATR-mediated signalling pathways. However in the absence of Chk1, such cells show a caspase-2-dependent apoptotic response that that bypasses Bcl-2, MOMP, and caspase-3 [31]. How caspase-2 triggers apoptosis is unclear as previous work suggests caspase-2 induces death through BID cleavage and MOMP [36]. In the p53-/-Chk1 depleted cells this death pathway is not activated (X) by IR.

Although deletion of Chk1 in mouse ES cells is lethal [26,27], many other cell types survive partial or complete loss of Chk1 function. Chk1-/- chicken DT40 cells remain viable although their growth is slowed (in part due to an increase in the level of spontaneous apoptosis, [28]). Additionally, Chk1 inhibitors are not uniformly toxic to cells at concentrations where Chk1 function is compromised and siRNA-mediated depletions of Chk1 do not invariably cause cell death [29-31]. However cells with a defective Chk1 response show increased sensitivity to wide range of DNA damaging agents and replication inhibitors. This is particularly evident in the form of an enhanced level of apoptosis in cells where Chk1 function is compromised by genetic knockouts, siRNA-mediated depletions or treatment with Chk1 inhibitors following exposure to agents that interfere with DNA replication [29-31] (Figure 1). This apoptotic response is caspase-3 dependent and appears to be initiated in cells in early S phase [29,30]. The onset of apoptosis is not rapid, Chk1 depleted cells become committed to death by 16 to 24 h after exposure to the replication inhibitors while Annexin V and caspase-3 induction occur somewhat later [32]. The response can be triggered in both p53 proficient and deficient cell lines. It was reported that primary human diploid fibroblasts treated with Chk1 inhibitors did not show this apoptotic response [29]. However there have been no further studies using primary cells and this should be confirmed with other cell types.

siRNA-mediated depletions of other DNA damage response proteins indicate that the apoptotic response is specific for cells defective in ATR-Chk1 signalling following disruption of DNA replication [33]. Tumour cell lines depleted of ATR showed a similar apoptotic response to replication inhibitors as the same cells depleted of Chk1 and co-depletion of ATR and Chk1 had no further effect. In contrast depletion of components of the ATM-mediated protein kinase cascade (ATM, Chk2, NBS1, or MRE11) had no significant effect on the apoptotic response to replication inhibitors. In addition, apoptotic responses of immortalized fibroblasts originating from individuals predisposed to cancer as a result of inherited mutations of ATM or NBS1 were not altered following treatment with replication inhibitors relative to cells corrected for these defects. Loss of the ATM-mediated signalling also does not alter the death response in ATR or Chk1-depleted cells.

These observations contrast with the central role played by the ATM-mediated protein kinase cascade in the apoptotic response to IR (Figure 1). ATM is central to the cellular response to DNA DSBs. In addition to its role in cell cycle checkpoints, this signalling cascade controls the onset of apoptosis following IR through the p53-mediated transcriptional activation of pro-apoptotic proteins such as BAX and PUMA [2-4]. Embryonic fibroblasts obtained form ATM-/-, Chk2-/-, NBS1-/- or p53-/- mice show defects in the induction of apoptosis by IR. MEFs deficient in ATM are partially defective while p53 deficient cells show a more complete resistance to the induction of apoptosis [34,35] although knockouts of both Chk2 and ATM show levels of apoptosis similar to those found in p53-/- cells [35]. Thus both ATM-dependent and independent pathways regulate the induction of apoptosis by IR.

While IR-induced apoptosis is suppressed in p53 deficient cells, recent work has shown that cell death can be restored by depletion of Chk1 [31] (Figure 1). A morpholino screen of p53 deficient zebrafish embryos revealed that radiation-induced apoptosis could be restored by knockdown of Chk1. Intriguingly this novel death pathway was caspase-2 dependent and was not affected by overexpression of bcl2/xl. In further contrast to the caspase-3-dependent pathway triggered by replication inhibitors in the absence of Chk1, the response to IR is ***dependent ***upon both ATM and ATR. This death response is conserved in human p53 deficient tumour cells. Depletion or inhibition of Chk1 in such tumour cells induced a caspase-2 dependent apoptotic response that was not detected in a p53 proficient line or in Chk1 depleted cells treated with replication inhibitors. How caspase-2 induces apoptosis is not clear as it is not an executioner caspase. Caspase-2 is thought to function upstream to cleave and activate BID that in turn causes mitochondrial outer membrane permeabilization, cytochrome c release, and activation of executioner caspases [36]. However this mitochondrial pathway does not appear to participate in the induction of death by IR in the absence of Chk1. Nevertheless, these findings reveal that Chk1 controls distinct cell death pathways triggered by DNA damage or perturbations of DNA metabolism.

### Mechanism(s)

Precise molecular events initiating apoptosis in the absence of Chk1 are not clear. Chk1 has a number of roles in cells in response to DNA replication stress. We have initially attempted to investigate this mechanism by determining which of these roles is most critical for the control of apoptosis. These studies indicate that the role of Chk1 in the suppression of inappropriate firing of replication origins is the primary determinant [37]. The main evidence for this is the suppressive effect of Cdc45 depletion on apoptosis in Chk1-depleted cells. Cdc45 is an essential co-factor for the replication helicase and a component of a protein complex involved in DNA replication initiation in human cells [38,39]. Although both of these processes may be affected by Cdc45 depletion, the effect of Cdc45 depletion on apoptosis [37]is only seen where inappropriate origin firing occurs in cells depleted of Chk1 [7,37]. Consistent with this hypothesis apoptosis is enhanced in cells defective in the negative effector of origin firing, p21, following Chk1 depletion and treatment with a replication inhibitor [30].

We speculated that stalled forks may generate a signal for cell death that is amplified when further replication forks are arrested as a result of the inappropriate origin firing in the absence of Chk1. A number of events associated with the inappropriate origin firing have been reported that could potentially provide such signals. Early after the inhibition of DNA replication in Chk1 depleted or inhibited cells, enhanced levels of RPA and γH2AX foci relative to controls cells can be detected [32,37]. Since the formation of these foci is also dependent upon the function of Cdc45, it is likely that this accumulation is a result of inappropriately fired origins in the absence of Chk1. At later times the RPA32 subunit of RPA is hyperphosphorylated. This is followed by a strong induction of γH2AX and persistent activation of ATM and Chk2 [32]. Despite the very high level of γH2AX seen in some of the cells (evident as a pan nuclear staining of γH2AX), these cells do not have detectable double strand breaks, consistent with previous reports for cells exposed to UV light [40]. Various reports have implicated H2AX phosphorylation state and the recruitment of proapoptotic cJun-N-terminal kinase (JNK1) after DNA damage [41,42]. Yet the Chk1-depleted cells showing the high levels of γH2AX are not committed to apoptosis. They can restart DNA synthesis if the replication inhibitor is removed but this does not resume at the sites of γH2AX foci and these cells do not appear to progress through S-phase [32]. Therefore, sites of γH2AX staining may represent abandoned replication forks that cannot be restarted.

RPA is phosphorylated by cells in S phase [43] but can be hyperphosphorylated at a number of sites in response to some types of DNA damaging agents (e.g. IR, UV, or MNNG, [44]) or DNA replication inhibitors [45]. Recent work indicates that hyperphosphorylated RPA co-localizes with the homologous recombination (HR) repair protein Rad51 at sites of ssDNA following treatment with a replication inhibitor and that the hyperphosphorylated form of RPA is required for HR and the maintenance of cell viability under these conditions [45]. Although it cannot yet be ruled out that the enhanced level of hyperphosphorylated RPA somehow assumes a different role in Chk1 depleted cells where HR is compromised [9], these data suggest that hyperphosphorylated RPA also contributes to cell survival rather than death. So, the identity of a putative cell death "signal transducer" remains elusive.

### Therapeutic applications

An exciting implication of this work is that tumour cells retain a cryptic apoptotic pathway that can be triggered by therapeutic replication inhibitors or even radiotherapy when Chk1 function is inhibited. Nevertheless the use of Chk1 inhibitors in therapy has not met with universal enthusiasm because of the important role played by Chk1 in the maintenance chromosomal stability and viability in dividing cells. A mouse strain in which Chk1 was specifically disrupted in adult mammary cells showed enhanced apoptosis and developmental defects [46]. Conditional Chk1 heterozygosity in these cells caused inappropriate cell cycle transitions and chromosomal abnormalities. Chk1 is highly expressed in many types of tumours [47] and this may protect them from replication stress induced by hypoxia or nutrient deprivation during tumour development [48] or the consequences of inappropriate transitions into S-phase triggered by genetic alterations acquired by tumour cells. The Chk1 knockdown experiments suggest enhanced lethality for tumour cells may be obtained where the protein is only transiently depleted, thus reducing potential lethality and genetic instability in normal tissue caused by complete loss of Chk1 or long term haploinsufficiency as in the conditonally heterozygous mice. Recent work has shown that Chk1 inhibitors can be used to increase the sensitivity of tumour cells to replication inhibitors *in vitro *and *in vivo *[49]. However initial studies using the nonspecific Chk1 inhibitor UCN-01 met complications in the form of a much increased half life in human serum due to an unfavourable association with human α-acid glycoprotein [50]. These difficulties may be overcome through the use of new and more specific Chk1 inhibitors [51] and several of these are already in phase I and II trails. It will be interesting to see whether the higher specificity of these agents improves the response.

An additional therapeutic application of Chk1 inhibitors was revealed by an siRNA screen for gene silencings synthetically lethal with Chk1 inhibition [52]. This screen identified genes required for Fanconi anemia pathway function as being particularly important for cell survival when Chk1 function was inhibited. Strikingly the effect of Chk1 inhibition and cisplatin in FA deficient cell lines was synergistic, suggesting that Chk1 inhibitors may be useful for the treatment of tumours containing mutations of FA genes or in combination with novel FA pathway inhibitors.

## Conclusions

Clearly much remains to be done to understand the mechanism(s) underlying Chk1 suppressed cell death. While findings thus far provide strong support for the use of Chk1 inhibitors in therapy, elucidation of mechanism(s) underlying Chk1 suppressed death may reveal novel therapeutic targets that may help overcome the resistance that frequently accompanies such targeted therapies.

## Conflict of interests

The author declares that he has no competing interests.
